# Codon-Pair Deoptimized (CPD) Intranasal RSV Vaccines: A Novel Strategy for Infant Protection

**DOI:** 10.3390/ijms27125231

**Published:** 2026-06-09

**Authors:** Wael Alturaiki

**Affiliations:** Department of Medical Laboratory Sciences, College of Applied Medical Sciences, Majmaah University, Al-Majmaah 11952, Saudi Arabia; w.alturaiki@mu.edu.sa

**Keywords:** RSV, intranasal vaccine, live-attenuated vaccine, CPD, mucosal immunity, infant immunization

## Abstract

Respiratory syncytial virus (RSV) is considered the leading causative agent of acute lower respiratory infections in infants and young children worldwide, which makes it a major contributor to pediatric morbidity and mortality. Infants are especially susceptible to severe disease in early life, which underlines the urgent need for developing effective immunization strategies against this virus. However, the development of vaccines against RSV has long been associated with significant challenges. For example, initial attempts, especially those involving formalin-inactivated RSV, resulted in vaccine-enhanced respiratory disease upon subsequent infection, which set a significant safety obstacle for future vaccine candidates. Other challenges facing vaccine development against RSV include the short-lived immunity induced by natural infection, lack of clear correlates of immunity, and immune naivety in infants. Recent breakthroughs in structural virology and immunology have provided insights into protective immunity against RSV, especially regarding neutralizing antibodies that recognize the virus in its prefusion conformation of the viral F protein. Among promising vaccine candidates, intranasal live-attenuated vaccines have emerged as especially promising for infant immunization, especially considering their close mimicry of natural infection that can elicit systemic as well as mucosal immunity in the respiratory tract. A newly emerging approach for live-attenuated virus vaccine development is codon-pair deoptimization (CPD), which is based on synthetic recoding that reduces viral replicative capacity while maintaining intact protein sequences and structure. The preclinical results of CPD-based RSV candidates have provided evidence of such vaccines’ ability to elicit robust immunity while maintaining favorable safety profiles. This review addresses the major challenges associated with the development of effective RSV vaccines for infant immunization, with particular emphasis on lessons learned from previous vaccine failures and recent advances in RSV vaccine development, particularly CPD-based attenuation strategies.

## 1. Introduction

Respiratory syncytial virus (RSV) is the major etiology of acute lower respiratory tract disease in young children and infants worldwide [1]. Globally, RSV is responsible for approximately 3.6 million childhood hospitalizations and around 100,000 childhood mortalities per year in children younger than 5 years of age [2]. Infants younger than 6 months are most vulnerable to serious RSV disease and mortality [2]. Thus, effective immunization is necessary.

However, the RSV vaccine effort has been historically hobbled by major challenges. In the 1960s, a formalin-inactivated RSV (FI-RSV) trial not only failed to provide protection in infants but also caused vaccine-enhanced disease, with approximately 80% of the vaccinated subjects hospitalized and two fatalities after subsequent RSV natural exposure [3,4,5]. The horrid outcome was the result of the failure of the vaccine to generate effective neutralizing antibodies or cytotoxic T-cell memory, in addition to an aberrantly balanced inflammatory Th2-biased response [6,7,8].

The FI-RSV incident set such vaccines back for decades, and safety became the major concern in the design of subsequent RSV vaccines. Given that natural RSV infection is fairly restricted within the respiratory mucosa and does not confer durable immunity, an effective RSV vaccine should generate strong mucosal immunity in the respiratory tract [9]. Mucosal immunization can stimulate the production of secretory IgA and tissue-resident T cells, both of which are critical for protective immunity at the site of infection. In this context, live-attenuated RSV vaccines delivered intranasally have emerged as particularly promising. These vaccines closely mimic natural infection, safely inducing broad humoral and cellular immunity with a low risk of vaccine-enhanced disease [10,11]. Notably, intranasal vaccine candidates have demonstrated the ability to elicit high levels of neutralizing antibodies and abundant mucosal IgA, both correlating with effective protection in challenge studies [12,13,14,15].

Another innovative approach for the generation of live-attenuated RSV vaccines is codon-pair deoptimization (CPD) [16]. CPD involves rewriting the viral genome in such a manner that the replication efficacy is reduced but not the viral protein amino acid sequence [16]. CPD has resulted in the development of highly attenuated but immunogenic RSV strains. For instance, the RSV-MinL4.0 candidate generates strong neutralizing antibody and T-cell responses equivalent to those in wild-type infection and provides complete protection in preclinical models [17]. This review provides an overview of vaccine clinical trial platforms for RSV, with an emphasis on the intranasal live attenuated vaccine utilizing a CPD-based strategy for infant immunization. Overall, the currently available preclinical and early clinical evidence supports the hypothesis that codon-pair deoptimization-based intranasal live-attenuated RSV vaccines represent a promising and rational strategy for infant RSV immunization.

## 2. RSV Classification, Genome, and Structure

RSV is a member of the family *Paramyxoviridae*, sub-family *Pneumovirinae*, and genus *Orthopneumovirus* [18]. RSV is a linear, single-stranded, negative-sense RNA virus with a genome of approximately 15.2 kb. The RSV genome encodes 10 genes that give rise to 11 proteins, including structural proteins required for viral assembly and non-structural proteins involved in viral replication and immune evasion [19,20,21]. The major structural proteins include the fusion protein (F), attachment protein (G), and small hydrophobic protein (SH), whereas the non-structural proteins NS1 and NS2 play critical roles in suppressing host immune responses [19,22].

**Figure 1:**
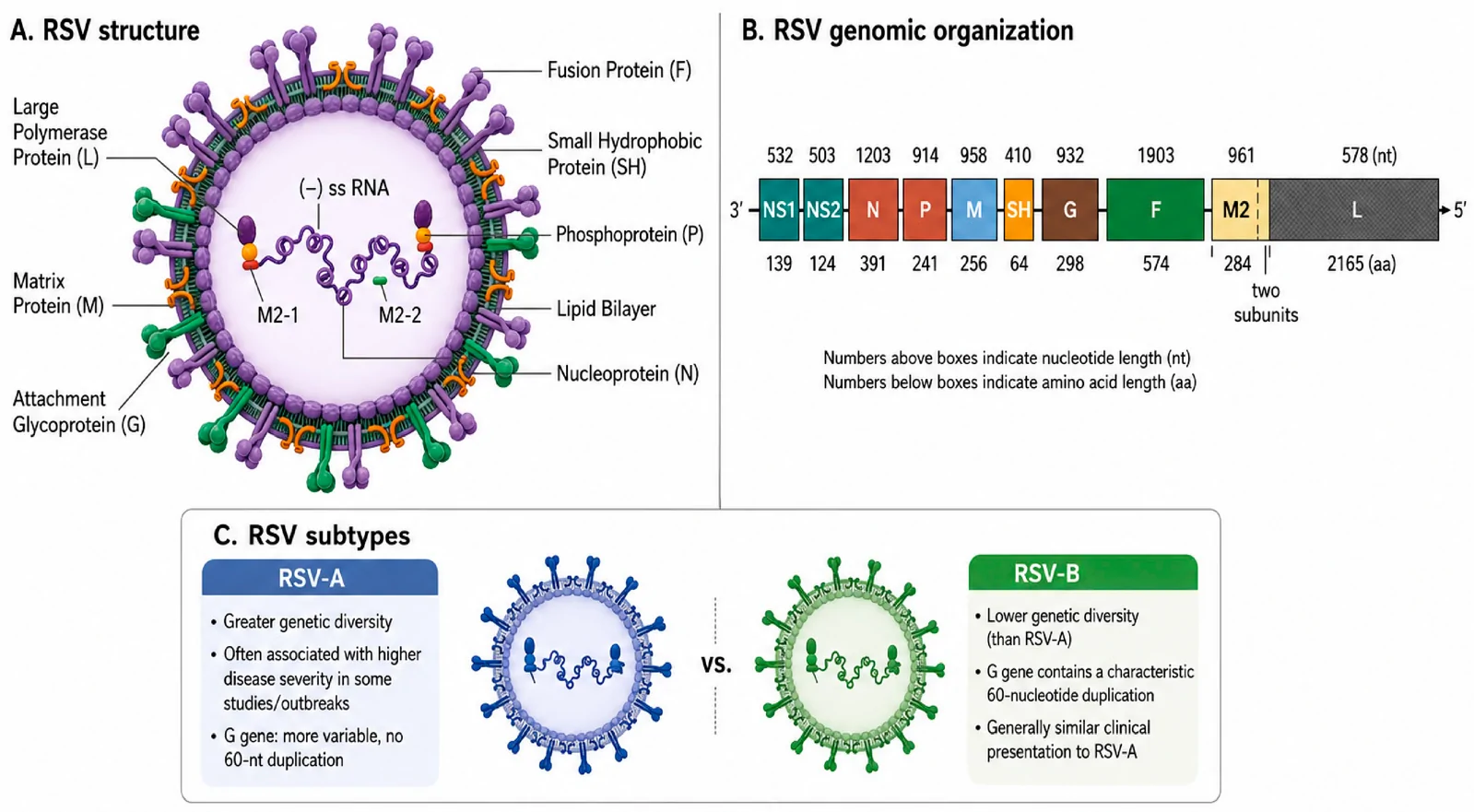
Structure and genomic organization of RSV and its subtypes. (**A**) Schematic representation of RSV virion structure. The viral envelope contains the attachment glycoprotein (G), fusion glycoprotein (F), and small hydrophobic protein (SH). The matrix protein (M) lines the inner surface of the envelope. The ribonucleoprotein complex consists of nucleoprotein (N), phosphoprotein (P), and large polymerase protein (L) bound to the negative-sense single-stranded RNA genome. The M2 gene encodes two regulatory proteins, M2-1 and M2-2. (**B**) Genomic organization of RSV in the 3′→5′ orientation. Numbers above the boxes represent nucleotide lengths; numbers below indicate amino acid lengths. (**C**) RSV is classified into two major antigenic subtypes, RSV-A and RSV-B, based primarily on sequence variability in the G glycoprotein gene. RSV-A strains are more genetically diverse and may be associated with greater disease severity in some studies, whereas RSV-B strains harbor a 60-nt duplication in the G gene. The F protein is highly conserved between the subtypes. The image was generated using ImageGen v2.0.

The RSV F protein mediates viral fusion and entry into host cells and represents the principal target for most current RSV vaccines because of its relatively conserved structure and ability to induce potent neutralizing antibodies. In contrast, the G protein facilitates viral attachment to host cell receptors and exhibits greater genetic variability, particularly between RSV subtypes, which may contribute to antigenic diversity and differences in disease epidemiology [19,20]. The RSV genome forms ribonucleoprotein (RNP) complexes that are essential for viral replication and transcription. Viral RNA is encapsidated by the nucleoprotein (N) in a helical structure that protects the genome from degradation by host cellular enzymes [23]. The phosphoprotein (P) and large polymerase protein (L) are also components of the RNP complex and facilitate RNA synthesis through interactions with the encapsidated RNA [24,25] (Figure 1).

The M2-1 and M2-2 proteins function as transcriptional regulators that promote efficient viral mRNA synthesis [26]. In addition, the RSV genome contains conserved 5′ and 3′ terminal regions that are essential for viral replication and transcription, as these regions contain promoter sequences recognized by the viral polymerase to initiate RNA synthesis [25,27]. The 5′ leader region also plays an important role in regulating genomic RNA packaging during virion assembly through sequence-specific interactions within the viral genome [28]. Notably, the absence of proofreading activity during RSV replication contributes to an increased mutation rate, thereby facilitating viral evolution and immune evasion [19,21].

RSV is classified into two major subtypes, RSV-A and RSV-B, based primarily on variation within the G protein gene, and both subtypes co-circulate during seasonal outbreaks [29]. Although the two subtypes share substantial genetic similarity, RSV-A has generally been associated with greater pathogenicity than RSV-B [30]. Because the F, G, and SH proteins are expressed on the viral surface, they represent important antigenic targets for vaccine development, with most current vaccine platforms focusing primarily on the F and G proteins to elicit effective neutralizing antibody responses [31,32]. Therefore, understanding the structural and functional organization of the RSV genome is important not only for elucidating viral pathogenesis but also for explaining the rationale behind current RSV vaccine strategies and the selection of viral targets involved in immunogenicity, immune evasion, and protective antibody responses.

## 3. Current and Emerging Strategies for RSV Prevention in Infants: Vaccine and Monoclonal Antibodies

Recent advances in RSV prevention have focused on the development of vaccines and monoclonal antibodies targeting the viral prefusion F (pre-F) glycoprotein, the major antigen responsible for inducing potent neutralizing antibodies. Prefusion F-based RSV vaccines, including Arexvy and Abrysvo, have received FDA approval for the prevention of RSV-associated lower respiratory tract disease in adults, whereas Abrysvo has additionally been approved for maternal immunization to protect infants through transplacental antibody transfer [33,34]. In addition, the mRNA-based RSV vaccine mRESVIA has been approved for adults aged 60 years and older [35].

### 3.1. Maternal Immunization Strategies for Early Infant Protection

Maternal immunization has emerged as one of the most promising strategies for protecting infants during the first months of life, a period characterized by high susceptibility to severe RSV disease and limited effectiveness of direct infant vaccination because of immune immaturity. Vaccination during pregnancy enables the transplacental transfer of RSV-specific maternal IgG antibodies, thereby providing passive protection during early infancy [33].

The bivalent prefusion F protein vaccine RSVpreF (Abrysvo, Pfizer) demonstrated significant efficacy in preventing medically attended severe RSV-associated lower respiratory tract illness in infants when administered during pregnancy between 32 and 36 weeks of gestation. In the phase III MATISSE trial, maternal vaccination reduced severe RSV-associated lower respiratory tract disease in infants by approximately 82% within the first 90 days after birth and maintained substantial protection throughout the first 6 months of life. Importantly, no major safety concerns were identified during the trial [33].

Maternal immunization provides several important advantages, including immediate protection at birth, reduced hospitalization risk during the first RSV season, and compatibility with existing maternal vaccination programs [36]. However, several limitations remain [37]. Protection is transient because maternally derived antibodies gradually decline during infancy, potentially leaving older infants susceptible to infection [38]. Placental antibody transfer may also be reduced in cases of preterm birth, malnutrition, or maternal comorbidities, particularly in low- and middle-income countries where RSV-associated mortality is highest [39]. Consequently, maternal immunization is likely to complement rather than replace active infant immunization strategies, including live-attenuated intranasal vaccines [40].

### 3.2. Monoclonal Antibodies for Passive RSV Immunoprophylaxis

Passive immunization with monoclonal antibodies represents another important strategy for protecting infants during their first RSV season. Currently approved monoclonal antibodies include palivizumab, nirsevimab, and clesrovimab [41,42,43]. While palivizumab is primarily reserved for high-risk infants, newer long-acting antibodies such as nirsevimab and clesrovimab provide broader seasonal protection through single-dose administration [43].

Clinical studies have demonstrated substantial efficacy of these preventive approaches in reducing severe RSV disease in young children. Maternal immunization with the bivalent prefusion F vaccine (Abrysvo) demonstrated 81.8% efficacy against severe medically attended RSV-associated lower respiratory tract disease within 90 days after birth and 69.4% efficacy within 180 days after birth in infants born to vaccinated mothers [33]. Similarly, the long-acting monoclonal antibody nirsevimab reduced medically attended RSV-associated lower respiratory tract infections by 70.1% and RSV-associated hospitalizations by 78.4% in healthy preterm infants during their first RSV season [42]. In high-risk infants, palivizumab prophylaxis reduced RSV-related hospitalization by approximately 55% [41]. These findings highlight the substantial clinical effectiveness of current RSV immunoprophylaxis strategies in reducing severe disease and hospitalization in young children. A comparison of the clinical efficacy of currently available RSV preventive strategies in infants and young children is summarized in Table 1.

Long-acting monoclonal antibodies have substantially transformed RSV prevention strategies for infants, particularly during the first RSV season [44]. Unlike vaccines, monoclonal antibodies provide immediate passive immunity without requiring the infant to generate an adaptive immune response, making them especially valuable for newborns and immunologically immature infants [45]. Palivizumab was the first licensed monoclonal antibody for RSV prevention; however, its use has been limited by the requirement for monthly dosing throughout the RSV season and its primary use in high-risk infant populations [46].

More recently, extended half-life monoclonal antibodies such as nirsevimab have demonstrated broader applicability in healthy term and preterm infants [47]. Clinical studies showed that a single dose administered before the RSV season significantly reduced medically attended RSV-associated lower respiratory tract infections and RSV-related hospitalizations throughout the RSV season [42]. These long-acting antibodies provide an important preventive option during the vulnerable early months of life and may substantially reduce the global burden of RSV-associated hospitalization [48]. Nevertheless, high manufacturing costs, distribution challenges, and limited accessibility in resource-limited settings remain important barriers to widespread implementation [49].

### 3.3. Current RSV Vaccine Candidates Under Clinical Development

In addition to currently approved RSV vaccines, several vaccine candidates using diverse vaccine platforms are under clinical investigation, including mRNA vaccines in lipid nanoparticles, live-attenuated vaccines, viral vector vaccines, protein subunit vaccines, and virus-like particle vaccines. The current clinical trial status and development phases of RSV vaccine candidates across different vaccine platforms are summarized in Table 2.

Although the success of mRNA vaccines in preventing infectious diseases such as COVID-19 is well recognized, their implementation for RSV prevention in infants remains limited because of safety concerns and the potential risk of vaccine-associated enhanced respiratory disease (VAERD). Recently, it has been reported that the FDA paused all RSV vaccine trials involving infants and RSV-naïve young children following an observed imbalance in severe RSV lower respiratory tract illness between vaccinated groups and controls. These findings renewed concerns regarding the historical FI-RSV-associated enhanced respiratory disease (ERD) observed in the 1960s and emphasized the importance of extensive safety evaluation and risk-management strategies in pediatric RSV vaccine development [50]. Consequently, alternative vaccine approaches are being explored, including rationally engineered live-attenuated RSV vaccines using CPD, which reduce viral replication while maintaining immunogenicity [51].

## 4. Intranasal RSV Vaccine Delivery: Advantages and Challenges

Intranasal vaccination represents an attractive strategy for RSV prevention because RSV infection is initiated at the respiratory mucosal surface. Unlike parenteral immunization, intranasal vaccines can induce both systemic and local mucosal immune responses, including secretory IgA antibodies and tissue-resident memory T cells within the respiratory tract. These immune responses are considered critical for limiting viral replication at the site of viral entry and may contribute to broader and more durable protection against RSV infection [52,53,54,55].

Live-attenuated intranasal RSV vaccines closely mimic natural infection while maintaining a favorable safety profile, particularly with respect to the reduced risk of vaccine-enhanced respiratory disease associated with FI-RSV vaccine [56]. Needle-free administration also offers practical advantages for pediatric immunization programs by improving patient compliance and simplifying vaccine delivery [57]. In addition, intranasal vaccination may reduce viral transmission by inducing mucosal immunity capable of limiting viral shedding in the upper respiratory tract [57].

Despite these advantages, several important challenges remain associated with intranasal vaccine delivery. Live-attenuated RSV vaccines generally require strict cold-chain storage conditions to maintain viral stability and viability, which may complicate distribution in low- and middle-income countries where RSV-associated mortality remains highest [58,59]. Additional concerns include variability in nasal administration efficiency, formulation stability, and the influence of pre-existing maternal antibodies on vaccine replication and immunogenicity in young infants [58]. Therefore, further optimization of intranasal vaccine formulations, thermostability, and delivery systems will be essential for large-scale global implementation [60,61,62].

## 5. Lessons from Past RSV Vaccine Failures

Early attempts to produce vaccines against RSV, especially the FI-RSV vaccine in the 1960s, identified fundamental immunologic pitfalls that have informed all subsequent vaccine development. FI-RSV induced high titers of antibodies with low neutralizing capacity and no CD8+ T-cell responses, but an exaggerated and unbalanced CD4+ T-cell response, which resulted in ERD and two deaths after the vaccinees were exposed to RSV [63,64]. Subsequent structural studies showed that FI-RSV primarily presents the post-fusion (Post-F) conformation of the RSV F glycoprotein, which lacks the prefusion (pre-F) glycoprotein epitopes that are the main target of potently neutralizing antibodies [64,65]. These findings highlighted that vaccine design can induce responses that, although non-protective, can be dangerous.

One of the most important lessons learnt is that RSV vaccine design should avoid vaccines that induce unbalanced and exaggerated CD4+ T-cell responses and low-quality, low-neutralizing antibodies in RSV-naive infants. Instead, vaccines that induce high-titer neutralizing antibodies, balanced T helper responses, and CD8+ T-cell responses to eliminate virus-infected cells and prevent immunopathology are required [65,66]. A second major lesson resulted from decades of live-attenuated vaccine research, which showed that, while many of these candidates were safe, they were too attenuated in older children and adults and therefore immunologically ineffective, demonstrating the narrow “therapeutic window” for attenuation [63,65,67]. The late-phase failures of subunit and vector vaccines further emphasized the need to better understand the immune response to RSV infection, including the lack of a definitive correlate of immunity, incomplete understanding of ERD risk, and the need to standardize immunological surrogates (such as neutralizing antibodies, multi-functional T-cells, and B-cell memory) across vaccine types and populations [63,64,68]. 

All of these vaccine failures have served to delay the field, but have directly led to the current pre-F-based vaccine candidates and mRNA/vector vaccine technologies that explicitly target prefusion F, seek to balance cell-mediated and humoral immunity, and have been evaluated with robust immunogenicity and ERD safety signals, especially in RSV-naïve infants and pregnant women [64,65,69,70]. Collectively, pharmacovigilance and challenge studies are refining these lessons, which emphasize the safety of vaccine-induced immunity in immunologically naïve individuals and the importance of antigen conformation as a key factor in RSV vaccine efficacy.

## 6. Codon-Pair Deoptimization (CPD): Concept and Mechanisms

**Figure 2:**
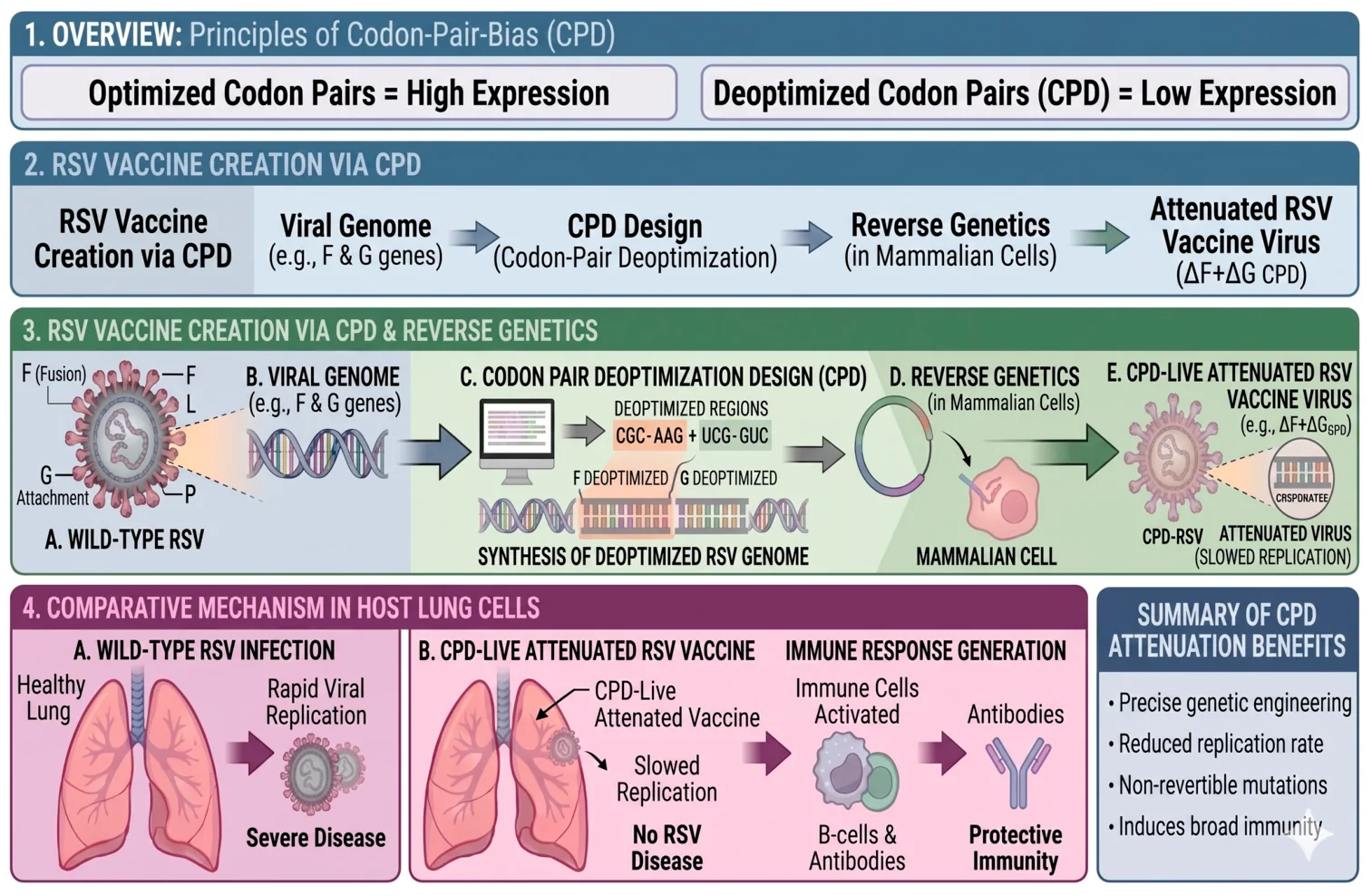
Overview of CPD–based development of a live-attenuated RSV vaccine. (1) The upper panel summarizes the principle of codon-pair bias, where optimized codon-pairs are associated with high gene expression, whereas CPD leads to reduced expression. (2) The second panel illustrates the general workflow for RSV vaccine creation via CPD, beginning with the viral genome (e.g., F and G genes), followed by codon-pair deoptimization design, reverse genetics in mammalian cells, and generation of an attenuated RSV vaccine virus. (3A–3E) The third panel provides a detailed schematic of CPD-mediated RSV vaccine development: (3A) wild-type RSV structure, (3B) viral genome selection, (3C) CPD and synthesis of the deoptimized RSV genome, (3D) reverse genetics and viral recovery in mammalian cells, and (3E) generation of a CPD-live attenuated RSV vaccine virus with slowed replication. (4A–4B) The fourth panel compares (4A) wild-type RSV infection, characterized by rapid viral replication and severe disease, with (4B) CPD-live attenuated RSV vaccination, which results in slowed replication, absence of severe disease, activation of immune cells, antibody production, and protective immunity. The right-side panel summarizes the advantages of CPD attenuation, including precise genetic engineering, reduced replication rate, non-revertible mutations, and broad immune induction. The image was generated using ImageGen v2.0.

CPD is a synthetic recoding strategy that exploits host-specific codon-pair bias to repress gene expression while preserving an unchanged encoded amino acid sequence (Figure 2). Codon-pair bias refers to the non-random frequency of adjacent synonymous codons in a genome, resulting from evolutionary selection acting on translational efficiency, mRNA stability, and nucleotide composition. The concept of CPD was first rigorously demonstrated in viral genomes by Coleman et al., 2008, who showed that genome-wide enrichment of suboptimal codon-pairs in poliovirus significantly attenuated viral fitness without altering the encoded proteins [51].

In CPD, viral open reading frames (ORFs) are subjected to synonymous recoding to favor the use of statistically suboptimal codon pairs, while maintaining amino acid identity. This technique has since been broadly used for the rational attenuation of RNA viruses for live vaccine applications [71]. CPD-mediated attenuation is principally influenced by reduced translational efficiency and reduced mRNA stability; importantly, Groenke et al. demonstrated that codon-pair-deoptimized influenza A virus segments show a notable decrease in transcript stability and protein expression, therefore offering convincing evidence for a direct mechanistic linking between unfavorable codon-pair context and diminished gene expression [72].

Moreover, CPD offers numerous advantages for vaccine design, including the maintenance of antigenic protein structure, lower reversion risk due to the high number of scattered synonymous substitutions, and adjustable attenuation by varying recoding intensity. CPD’s successful application across multiple RNA viruses demonstrates its versatility as a synthetic attenuation tool [51,71,72]. Collectively, mechanistic and experimental evidence supports CPD as a rational, efficient, and genetically stable strategy for the advancement of live-attenuated viral vaccines.

Currently, there is no licensed actively immunizing RSV vaccine administered directly to infants, although maternal vaccination and long-acting monoclonal antibodies are now available for infant protection. This is mainly because of the challenges associated with achieving the appropriate balance between safety and immunogenicity of the conventional live attenuated RSV vaccines under development. Genome recoding of the virus, particularly CPD, has been developed to circumvent the challenges associated with artificial attenuation of the virus for immunization purposes [73].

## 7. Challenges in the Development of Direct RSV Vaccines for Young Infants

The development of a direct vaccine for RSV targeting young infants has encountered numerous connected challenges across multiple fields, including virology, immunology, safety, trial design, and implementation. A significant challenge is the inadequate understanding of protective immunity during early life, as the natural RSV infection results in limited protection and ineffective immunity, leading to frequent reinfections and a lack of consensus on correlates of protection, despite the involvement of neutralizing antibodies, mucosal IgA, and cell-mediated responses [68]. This makes it difficult to define target immune responses and to benchmark infant vaccines. The historical experience of enhanced respiratory disease (ERD) following the 1960s FI-RSV vaccine in RSV-naive infants, which led to severe lung inflammation and deaths on subsequent natural infection, has imposed a very high safety bar; regulators require extensive preclinical and staged clinical data to exclude ERD before large trials in naïve infants can proceed [68,74,75].

Designing vaccines that induce balanced, Th1-skewed immune responses without triggering ERD-associated Th2 immunopathology remains particularly challenging in immunologically immature infants. In addition, viral factors further complicate antigen design, as the RSV F protein is structurally dynamic and only its prefusion conformation exposes highly potent neutralizing epitopes. Stabilizing this conformation and preserving it throughout manufacturing and storage therefore require extensive structure-based engineering efforts [74,76,77].

In practice, the period of greatest disease risk is the first 3 to 6 months of life, which coincides with immunological immaturity and the persistence of maternally produced antibodies, both of which may restrict the efficacy of active infant immunization. As a result, most current preventive efforts prioritize maternal vaccination or monoclonal antibody delivery over direct immunization of infants in early life [77,78,79,80]. Vaccine candidates designed for usage as early as 6 months of age, such as live-attenuated and viral vector platforms, must strike an appropriate balance between attenuation for safety and adequate replication to produce protective immune responses. Notably, at least one adenoviral-vectored candidate did not show the expected efficacy in newborns, underscoring the uncertainties associated with specific platforms [68,80,81].

Furthermore, there are significant logistical and ethical obstacles associated with conducting large, placebo-controlled efficacy trials in very young infants. Such investigations necessitate well-defined and consistent clinical outcomes, which remain a subject of debate and may differ depending on age group and epidemiological setting [68]. If a safe and effective newborn vaccine is created, providing equal global access will remain a top priority. Over 95% of RSV-related deaths occur in low- and middle-income countries, where vaccine cost, cold-chain capabilities, and integration into current maternal and child health programs will ultimately decide real-world efficacy [82].

### 7.1. Application of CPD in Advancing Intranasal RSV Vaccines for Infants

#### 7.1.1. Preclinical Development and Immunogenicity

Preclinical development of CPD-attenuated RSV vaccines has demonstrated a rational strategy for achieving viral attenuation while preserving immunogenicity. The application of CPD to RSV enabled the generation of multiple recoded RSV variants (Min A, Min B, Min L, and Min FLC) through genome-scale synonymous recoding of viral open reading frames [73].

These CPD viruses exhibited reduced replication efficiency in vitro, temperature sensitivity, and attenuation in vivo in both mice and African green monkeys, while maintaining antigenic integrity due to preservation of the amino acid sequence.

Building on this foundation, further optimization of CPD RSV candidates led to the development of more refined constructs, including RSV-MinL4.0, which was evaluated in a non-human primate model. In African green monkeys, RSV-MinL4.0 demonstrated strong attenuation, characterized by markedly reduced viral replication and minimal virus shedding following intranasal administration. Importantly, vaccination induced robust humoral and cellular immune responses and conferred protection against viral replication following wild-type RSV challenge, supporting its translational potential [17].

Recently, advanced CPD RSV candidates have been engineered by increasing the number of recoded genomic regions to enhance attenuation and genetic stability. The Min AL construct, generated by codon-pair deoptimization of seven viral open reading frames, represents a next-generation candidate with over 2000 synonymous mutations. In hamster models, Min AL exhibited highly restricted replication in both the upper and lower respiratory tracts, while inducing strong systemic (IgG) and mucosal (IgA) immune responses comparable to those elicited by wild-type RSV infection. Furthermore, intranasal immunization with Min AL conferred complete protection against viral challenge, highlighting its potential as a safe and effective live-attenuated vaccine candidate [16].

Overall, these studies show that CPD-based RSV vaccines are effective in finding the appropriate balance between attenuation, stability, and immunogenicity. Notably, the capacity of the CPD-based RSV vaccines to induce mucosal and systemic immunity after intranasal immunization represents a significant advantage for infant immunization because the development of mucosal immunity is critical for the control of RSV infection in the respiratory tract.

#### 7.1.2. Clinical Trials and Pediatric Evaluation

CodaVax-RSV vaccine is a promising vaccine candidate that is currently undergoing clinical trials (ClinicalTrials.gov Identifier: NCT04919109), which is a live-attenuated vaccine that targets RSV. This vaccine was designed through the application of the CPD approach to the entire RSV genome. This Phase 1 randomized double-blind placebo-controlled dose-escalating study aims to assess the safety and immunogenicity of the vaccine in a group of healthy RSV-naive and RSV-experienced children between the ages of 6 months and 5 years. The primary objectives of this study are to assess the reactogenicity of the vaccine as well as the immune response. Furthermore, the CodaVax-RSV vaccine has been awarded Fast Track status by the FDA. This demonstrates that the vaccine has the potential to address a significant unmet medical need in the prevention of RSV infection in the pediatric population [83]. This study signifies a major first step in assessing the viability of CPD-based attenuated vaccines for infants and toddlers, who are one of the most susceptible cohorts to severe RSV infection. Furthermore, though CPD has proven to be extremely promising as a vaccine adjuvant, its efficacy in licensed vaccines targeting RSV infection in children must undergo a detailed safety assessment, especially in seronegative children, where the possibility of immunopathogenesis is a major cause of concern. Optimized versions of the CPD construct and the positive results of such early-stage studies as NCT04919109 could mark a revolutionary leap in the field of RSV vaccines.

## 8. Conclusions and Future Perspectives

RSV remains a major cause of respiratory disease in infants and young children, and the development of safe and effective vaccines continues to present significant scientific and clinical challenges. Past failures of RSV vaccines have highlighted the importance of inducing protective immune responses while maintaining vaccine safety. Recent advances in structural virology and immunology have improved understanding of RSV immunity and supported the development of novel vaccine platforms. Intranasal live-attenuated RSV vaccines are considered particularly promising because they mimic natural infection and induce both mucosal and systemic immune responses in the respiratory tract.

CPD represents a rational strategy for RSV attenuation because it reduces viral replication while preserving viral protein sequences and antigenicity. Preclinical studies have demonstrated that CPD-based RSV vaccines can induce protective immune responses with favorable safety profiles. Importantly, the available evidence supports the central hypothesis that CPD-based intranasal live-attenuated RSV vaccines represent a promising strategy for infant RSV immunization. Experimental and preclinical studies consistently demonstrate that CPD can achieve effective viral attenuation while maintaining antigenicity and inducing protective mucosal and systemic immune responses. However, the efficacy of CPD vaccine candidates appears to depend on achieving an optimal balance between attenuation and immunogenicity. Candidates with excessive codon-pair deoptimization may become over-attenuated and generate weaker immune responses, whereas moderately attenuated constructs may retain sufficient replication to induce stronger immunity. In addition, preservation of antigenic protein structure is likely critical for effective immune protection. Potential limitations of CPD-based vaccines include variable attenuation efficiency across different host cells and species due to differences in codon-pair preferences and translation machinery, which may affect viral replication and immunogenicity; variable immune responses in RSV-naive infants, particularly in the presence of maternal antibodies; and the need for continued evaluation of long-term genetic stability and safety. Furthermore, the successful implementation of intranasal live-attenuated RSV vaccines may also depend on overcoming important formulation and delivery challenges. Because live-attenuated vaccines generally require strict cold-chain storage conditions to preserve viral stability and infectivity, maintaining vaccine potency during transportation and storage may be particularly difficult in low- and middle-income countries, where the burden of RSV-associated mortality is highest. Additional considerations include optimization of intranasal delivery devices, formulation thermostability, dosing consistency, and the potential influence of maternally derived antibodies on vaccine replication and immunogenicity in young infants. Therefore, improving the stability and practicality of intranasal vaccine delivery systems will likely be essential for large-scale global implementation of CPD-based RSV vaccines.

Although CPD introduces a large number of synonymous mutations that reduce the likelihood of direct reversion to the wild-type sequence, the theoretical risk of compensatory mutations restoring viral fitness cannot be completely excluded and requires further investigation.

## Figures and Tables

**Table 1:** Clinical efficacy of current RSV preventive strategies in infants and young children.

Preventive Strategy	Target Population	Clinical Outcome	Efficacy/Reduction	Reference
Maternal RSVpreF vaccine (Abrysvo)	Infants born to vaccinated mothers	Severe medically attended RSV-associated lower respiratory tract disease within 90 days after birth	81.8% efficacy	[33]
Maternal RSVpreF vaccine (Abrysvo)	Infants born to vaccinated mothers	Severe medically attended RSV-associated lower respiratory tract disease within 180 days after birth	69.4% efficacy	[33]
Palivizumab	High-risk infants	RSV-associated hospitalization	~55% reduction	[41]
Nirsevimab	Healthy preterm infants during the first RSV season	Medically attended RSV-associated lower respiratory tract infection	70.1% reduction	[42]
Nirsevimab	Healthy preterm infants during the first RSV season	RSV-associated hospitalization	78.4% reduction	[42]
Clesrovimab	Healthy infants during the first RSV season	Medically attended RSV-associated lower respiratory tract infection	60.4% reduction	[43]
Clesrovimab	Healthy infants during the first RSV season	RSV-associated hospitalization	84.2% reduction	[43]

**Table 2:** Current and ongoing clinical trial status for multiple RSV vaccine candidates among various vaccine platforms. Some have received FDA approval, whereas others remain in the clinical trial phase.

Platform Type	Trial ID	Vaccine Candidate	Manufacturer	Specific Antigen	Target Population	Phase/Status
mRNA (LNP)	NCT06143046	mRNA-1345 (maternal)	Moderna	Prefusion RSV F (RSV-A)	Pregnant women	Approved by the FDA
	NCT05743881	mRNA-1345 (pediatric)		Prefusion RSV F	Infants/children (5–24 mo)	Suspended due to safety concerns
	NCT05639894	LNP CL-0059/CL-0137	Sanofi Pasteur	Unspecified RSV antigen	Healthy adults (18–50 and ≥60 yr)	Phase I/IIa
	NCT06287450	IN006	Shenzhen Shenxin (INNORNA)	Prefusion F (RSV-A and RSV-B)	Adults ≥ 18 yr (China)	Phase I/II
	NCT05585632	mRNA-1045	Pfizer/BioNTech	Influenza HA + RSV preF	Adults (50–75 yr)	Phase I
Live-attenuated	NCT06252285	RSV ANS2/A1313/11314L (“RSVt”)	Sanofi	Whole RSV	Infants/toddlers (6–21 mo)	Phase III (terminated)
	NCT04690335	MV-012-968	Meissa Vaccines	Whole RSV ΔNS1/ΔNS2/ΔG/ΔSH virus	Adults (challenge model)	Phase IIa
	NCT04295070	CodaVax-RSV (adults)	Codagenix	Whole RSV	≥18 yr	Phase I
	NCT04919109	CodaVax-RSV (peds)			Children (6 mo–5 yr)	Phase I
Viral Vector	NCT03213405	rBCG-N-hRSV	IDT Biologika/Chile	RSV Nucleoprotein (N)	Adults (18–50 yr)	Phase I
	NCT03473002	SeVRSV	St. Jude/Serum Institute	RSV Fusion (F) protein	Adults (18–45 yr)	Phase I
	NA	rVSV-G-2A-F	Academic/OSU	RSV G + F proteins	Preclinical	Preclinical
	NCT05281263	BLB-201 (PIV5)	Blue Lake Biotech	Full-length RSV F	Adults (18–59 yr);	Phase I
	NCT05655182				Children (8–59 mo)	Phase I/I–IIa
	NCT05070546	Ad26.RSV.preF	Janssen (J&J)	Prefusion RSV F	Adults ≥ 65 yr	Phase III
	NCT05238025	MVA-RSV	Bavarian Nordic	RSV F, G, N, M2-1	Adults ≥ 60 yr	Phase III (Failed)
Protein Subunit	NCT04681833	BARS13	Advaccine (Suzhou)	RSV G protein	Elderly (60–80 yr)	Phase II
	NCT06194318	SCB-1019	Clover Biopharma	Prefusion F (RSV-A/B)	Adults (60–85)	Phase I
	NCT04519073	SCB-1019	Virometrix AG	RSV F antigenic site II	Adults (18–45)	Phase I
	NCT04886596	Arexvy	GlaxoSmithKline	Prefusion RSV F (AS01E adjuvanted)	Adults ≥ 60	Approved by the FDA
	NCT04424316	Abrysvo	Pfizer	Bivalent Prefusion F (RSV-A and RSV-B)	Adults ≥ 60; Pregnant women	Approved by the FDA
Nanoparticle (VLP)	NCT05655689	IVX-121	Icosavax	Prefusion RSV F (multivalent nanoparticle display)	Adults (60–85)	Phase I
	NCT05903183	IVX-A12	Icosavax/AstraZeneca	Prefusion RSV F + hMPV F	Adults (60–85)	Phase IIa
	NCT02624947	ResVax	Novavax	Post-fusion RSV F protein nanoparticle	Pregnant women	Phase III (Maternal trial failed; program discontinued)
	NCT02608502				Older adults ≥ 60	

## Data Availability

No new data were created or analyzed in this study. Data sharing is not applicable to this article.
